# Occurrence and clinical management of moderate-to-severe adverse events during drug-resistant tuberculosis treatment: a retrospective cohort study

**DOI:** 10.1186/2052-3211-7-14

**Published:** 2014-10-21

**Authors:** Evans L Sagwa, Aukje K Mantel-Teeuwisse, Nunurai C Ruswa

**Affiliations:** School of Public Health, University of the Western Cape, Cape Town, South Africa; Utrecht Institute for Pharmaceutical Sciences, Division of Pharmacoepidemiology and Clinical Pharmacology, Utrecht University, Utrecht, the Netherlands; National Tuberculosis and Leprosy Programme, Ministry of Health and Social Services, Windhoek, Namibia

**Keywords:** Medication safety, Second-line anti-tuberculosis drugs, Pharmacovigilance, Adverse effects, TB/HIV co-infection, Namibia

## Abstract

**Objectives:**

To determine the incidence of symptomatic moderate-to-severe adverse events during treatment of drug-resistant tuberculosis, and to compare their risk and outcomes by patients’ human immunodeficiency virus (HIV) co-infection status.

**Methods:**

We conducted a retrospective cohort analysis of patients treated for drug-resistant tuberculosis between January 2008 and February 2010. Routinely, clinicians monitored and managed patients’ response to treatment until its completion. Any symptomatic adverse event observed by the clinician or reported by the patient was recorded in the standard patient treatment booklet of the National Tuberculosis and Leprosy Programme. There were 18 symptomatic adverse events routinely monitored. Depending on the nature of the medical intervention needed, each was graded as mild, moderate or severe. Data were extracted from the patient treatment booklet using a structured form, then descriptive, bivariate and Cox proportional hazard analysis performed, stratified by patients’ HIV infection status. Statistical associations were done at the 5% level of significance and reported with 95% confidence intervals.

**Results:**

Fifty seven (57) patients with drug-resistant tuberculosis were identified, 31 (53%) of whom were HIV co-infected. The cumulative incidence of moderate-to-severe adverse events was 46 events in 100 patients. HIV co-infected patients experienced more moderate-to-severe adverse events compared with the HIV uninfected patients (median 3 versus 1 events, *p* = 0.01). They had a four-fold increase in the cumulative hazard of moderate-to-severe adverse events compared with the HIV uninfected patients (HR = 4.0, 95% CI 1.5 – 10.5). Moderate-to-severe adverse events were the main determinant of a clinician’s decision to reduce the dose or to stop the suspected offending medicine (RR = 3.8, 95% 1.2-11.8).

**Conclusions:**

Moderate-to-severe adverse events are common during drug-resistant tuberculosis therapy. They are more likely to occur and to persist in HIV co-infected patients than in HIV uninfected patients. Clinicians should employ various strategies for preventing drug-induced patient discomfort and harm, such as reducing the dose or stopping the suspected offending medicine. Managers of tuberculosis control programmes should strengthen pharmacovigilance systems. We recommend a more powered study for conclusive risk-factor analysis.

**Electronic supplementary material:**

The online version of this article (doi:10.1186/2052-3211-7-14) contains supplementary material, which is available to authorized users.

## Introduction

The burden of tuberculosis (TB) disease in Namibia remains high, with a case notification rate of 545 cases per 100,000 population in 2012 [1]. The prevalence of drug-resistant tuberculosis (DR-TB) in the country, estimated at 20.1 cases per 100,000 TB patients, combined with the high human immunodeficiency virus (HIV) co-infection rate of about 50%, is a major public health concern for the National Tuberculosis and Leprosy Programme (NTLP) [1].

Both DR-TB and HIV infections need to be treated, otherwise, the patient may not survive for too long [2, 3]. The adverse effects of second-line anti-tuberculosis and antiretroviral medicines pose a unique challenge in the combined treatment of the two infections [2, 3]. Moderate-to-severe adverse events can cause patients’ intolerance to second-line anti-tuberculosis medicines and antiretroviral medicines, possibly compromising DR-TB and HIV treatment outcomes. Such intolerance may require the clinician treating the patient to make specific medicine dosage adjustments, regimen changes or stop the treatment [4, 5]. Similarly, treatment of HIV with highly active antiretroviral therapy (HAART) is associated with various adverse effects, some of which may overlap with those of second-line anti-tuberculosis medicines [2, 3, 6].

This paper is the third and last of a series of papers [7, 8] that we have published based on a dataset on the occurrence of adverse events during treatment of DR-TB in Namibia, each paper addressing a different aspect of the adverse events epidemiology. The first paper described the burden of adverse events during treatment of DR-TB, [7] while the second paper compared, by HIV co-infection status, the risks and the risk-factors for the commonly observed adverse events [8]. Apart from our research highlighted above, there is limited scientific literature on the incidence, clinical management and the outcomes of moderate-to-severe adverse events among patients on DR-TB therapy in Namibia.

In this paper, we describe the cumulative incidence and the actions taken by clinicians to manage the moderate-to-severe adverse events occurring during DR-TB treatment. Secondly, we compare the risk and outcomes of these moderate-to-severe adverse events, by patients’ HIV co-infection status.

## Methods

### Study design

This was a retrospective observational cohort study of consecutive patients treated for DR-TB between January 2008 and February 2010 at the Kondja DR-TB treatment ward in the Walvis Bay District of Namibia. All the DR-TB patients treated at this facility during the specified period were included in the study.

### Setting

The study was conducted at the Kondja DR-TB treatment ward, which is a 25-bed district hospital DR-TB treatment facility serving the entire Erongo region of Namibia. The Erongo region had the second largest number of patients on DR-TB treatment in Namibia at the time of the study. In this ward, patients with microbiologically confirmed DR-TB infection were placed on second-line intensive phase treatment that included parenteral amikacin, kanamycin or capreomycin for a minimum of four months, until two sputum smears and two successive cultures turned negative [7, 9]. Clinicians designed individualized regimens and calculated daily doses of each medicine based on patients’ body weight, in accordance with the national TB treatment guidelines published in 2006 [9]. The HIV co-infected patients were, additionally, treated with HAART regimens that comprised of lamivudine in combination with either zidovudine (AZT) or stavudine (d4T) and efavirenz (EFV) or nevirapine (NVP) [9].

The susceptibility of *M. tuberculosis* to anti-TB medicines was tested by the Namibia Institute of Pathology using the liquid culture MGIT 960 system (BACTEC™ MGIT™ 960 Mycobacteria Culture System, Becton Dickinson, New Jersey, USA) on all *M. tuberculosis* confirmed cultures, for susceptibility to isoniazid, rifampicin, streptomycin and ethambutol. All isolates of *M. tuberculosis* found to be resistant to rifampicin or isoniazid were sent to the National Health Laboratory Service in South Africa for testing of resistance to kanamycin, capreomycin, amikacin, ciprofloxacin, levofloxacin and ethionamide.

Routinely, during DR-TB treatment, patients were closely monitored and supervised by the clinician until completion of treatment. Any clinician-observed or patient-reported symptomatic adverse events were recorded in the standard patient treatment booklet designed by the NTLP. At the time of the study, the DR-TB patient treatment booklet listed 18 symptomatic adverse events that were routinely monitored during treatment: abdominal pain, constipation, hearing loss (decreased hearing), depression, diarrhoea, dizziness, fatigue, fever, headache, joint pain, nausea, neuropathy, psychosis, rash, tinnitus, tremors, vision changes and vomiting [9]. According to the DR-TB patient treatment booklet (Additional file 1), the severity of an adverse event could be classified into three grades. Grade 1 were the mild adverse events, requiring no medical intervention; Grade 2 were the moderate adverse events, requiring palliative [or adjunctive] intervention; while Grade 3 were the severe adverse events, requiring a change in treatment or its discontinuation [9]. Each observed adverse event was graded by the attending clinician as mild, moderate or severe as explained above and was managed according to the severity grading.

### Ethical considerations

Ethical approval of the study protocol was obtained from the research unit of the Ministry of Health and Social Services of Namibia (MoHSS) – Ref 17/3/3/AP and the Higher Degrees Committee of the University of the Western Cape, South Africa, both of which are institutional review boards.

### Data collection

The lead researcher (corresponding author) collected data from patients’ DR-TB treatment booklet using a structured form. No personal identifiers were recorded, to maintain the anonymity and the confidentiality of the patients. The primary study outcome was the occurrence of any adverse event during DR-TB treatment. The secondary outcome was the occurrence of moderate-to-severe adverse events. Further, detailed characterization of each moderate-to-severe adverse event was conducted, which included: its description, time-to-onset, severity grading, duration, actions taken to manage the adverse event, and the outcome of the adverse event.

### Definition of terms

In this study, DR-TB included both poly- and multidrug resistant forms of *M. tuberculosis*. Poly drug-resistance was defined as the resistance of *M. tuberculosis* to either isoniazid or rifampicin and other first-line anti-tuberculosis medicines, while multidrug resistance was the resistance to at least both isoniazid and rifampicin [9].

### Data analysis

We limited our statistical analyses to descriptive and univariate analysis, due to the small sample of DR-TB patients that was realized. We couldn’t perform multivariable analyses because of the few degrees of freedom of the small sample. We therefore calculated absolute and relative frequency counts, measures of central tendency (mean and median) and measures of dispersion including range, interquartile range and standard deviation. We applied two-tailed Student’s T-tests to compare group differences in age and weight after testing for normality. For non-normally distributed variables such as the number of adverse events observed, comparisons were made by the non-parametric Mann–Whitney/Wilcoxon two sample test. We compared proportions and categorical variables using the Chi-square or Fisher exact test respectively, depending on whether or not the expected value for a cell in the cross-tabulation was greater than five.

Associations between exposure and outcome variables were assessed using 2×2 contingency tables, with further stratification by HIV infection status. In addition, Kaplan-Meier and Cox proportional hazard analysis were performed to generate hazard ratios. All the analyses were done in Epi Info 3.4.3. (November 2007, Centers for Disease Control and Prevention, Atlanta, USA) and reported as point estimates, 95% confidence intervals (95% CI) and *p*-values. However, the Kaplan-Meier plot was drawn using the Statistical Package for the Social Sciences (SPSS®) for Windows, version 12.0.1 (IBM Corporation, New York, USA). A *p*-value of less than 0.05 was considered to be statistically significant. Lastly, we used Microsoft Excel® (Microsoft office 2010, Microsoft Corporation, Redmond, Washington State, USA) to draw charts and tables.

## Results

**Figure 1:**
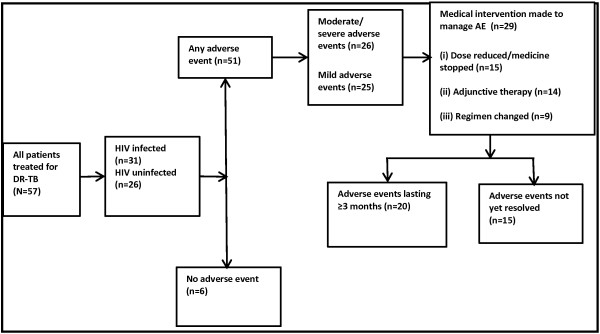
**Flow diagram of DR-TB treatment, occurrence and outcomes of adverse events.** Legend for Figure 1: DR-TB = drug resistant tuberculosis; HIV = Human immunodeficiency virus; AE = adverse event.

The proportion of DR-TB patients who experienced any adverse event was 51/57 (89%). Of these 51 patients, 26 (51%) experienced at least one moderate-to-severe adverse event. A medical intervention was made to manage the adverse event in 29 (57%) of the patients. These medical interventions included reducing the medicine dose or stopping the suspected offending medicine in 15 patients (29%), using other adjunctive medicines to treat the adverse event(s) in 14 patients (27%), or completely changing the DR-TB treatment regimen in 9 patients (18%). There were 20/51 (39%) patients who experienced persistent adverse events that lasted for three months or more, while 15/51 (29%) patients were yet to recover from their adverse events by the study end date (Figure 1).

The distribution of the patients’ demographic and clinical characteristics was generally similar between the 26 DR-TB patients who experienced at least one moderate-to-severe adverse event compared to the 25 who experienced at least one mild adverse event (Table 1). However, the HIV co-infection rate was notably higher among the patients who experienced at least one moderate-to-severe adverse event, compared with those who experienced only mild adverse events (69.2% versus 40%, *p* = 0.04).

**Table 1:** **Demographic and clinical characteristics of the patients, by adverse event severity grading**

	Adverse events by severity grading (N = 51)		
	Moderate-to-severe events (n = 26)	Mild events (n = 25)	***P***-value
Gender: Male, n (%)	16 (61.5%)	15 (60.0%)	0.91
Age: mean ± SD, yrs	34.1 ± 8.3	35.0 ± 10.2	0.71
Weight: mean ± SD, kg	51.4 ± 10.3	53.9 ± 12.3	0.45
HIV co- infection, n (%)	18 (69.2%)	10 (40%)	0.04
HAART, n (%)	5 (19.2%)	7 (28%)	0.46
Duration (days) of therapy; median (IQR)	183.5 (173–243)	185 (175.5-212)	0.81
Number of drugs in intensive phase regimen; median (IQR)	5 (5–6)	5 (5–6)	0.61

SD = standard deviation; yrs = years; kg = kilogrammes; HIV = human immunodeficiency virus; HAART = highly active antiretroviral therapy; TB = tuberculosis; IQR = interquartile range.

**Figure 2:**
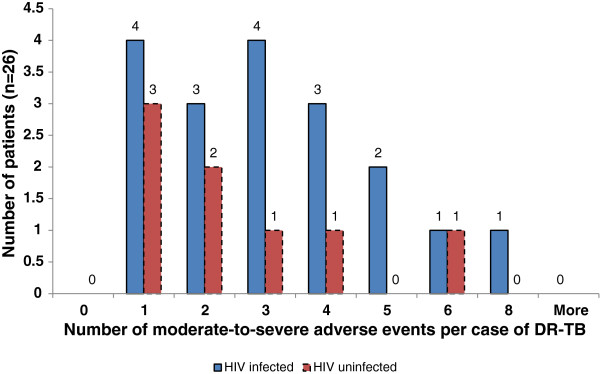
**Distribution of the number of moderate-to-severe adverse events by HIV infection status.** Legend for Figure 2: DR-TB = drug resistant tuberculosis.

Overall, the DR-TB patients co-infected with HIV experienced more moderate-to-severe adverse events compared with the HIV uninfected patients, with a median of 3 adverse events versus 1 adverse event respectively (*p* = 0.01), as depicted in Table 2. Eighteen of the 26 DR-TB patients who experienced at least one moderate-to-severe adverse event (69%), were HIV co-infected (Figure 2).

**Table 2:** **Frequency of moderate-to-severe adverse events by HIV infection status**

	Frequency of moderate-to-severe adverse events	
Adverse events	HIV infected	HIV uninfected
Tinnitus	9	6
Joint pain	7	0
Decreased hearing	8	4
Nausea	5	1
Headache	3	1
Fatigue	5	1
Abdominal pain	5	1
Dizziness	3	0
Rash	3	0
Vomiting	3	0
Diarrhea	2	1
Neuropathy	2	1
Fever	0	0
Vision changes	0	2
Depression	1	0
Psychosis	1	1
Tremors	1	1
Constipation	0	0
**Total number of adverse events**	**58**	**20**
**Median number of adverse events**	**3**	**1**

Difference in median (3–1) = 2. *p* = 0.01 (Mann–Whitney/Wilcoxon Two-Sample Test).

The cumulative incidence of moderate-to-severe adverse events in the entire cohort was 26 events out of 57 patients (46 events in 100 patients). By comparison, the cumulative incidence of moderate-to-severe adverse events amongst the HIV co-infected patients was 18 events out of 28 patients (64 events in 100 patients) while it was 8 events out of 23 patients (35 events in 100 patients) amongst the HIV uninfected patients, (*p* = 0.04).

**Figure 3:**
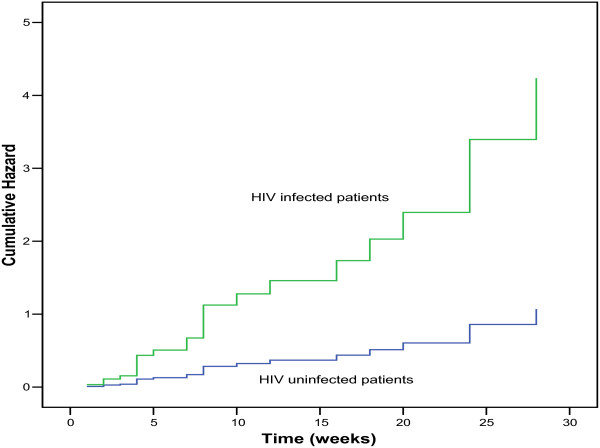
**Kaplan Meier curve of the time to the occurrence of moderate-severe adverse events, by HIV status.** Legend for Figure 3: HIV = Human immunodeficiency virus.

In a time-to-event analysis using a Kaplan Meier curve and Cox proportional hazards analysis, the DR-TB patients who were co-infected with HIV had a four-fold cumulative hazard of experiencing moderate-to-severe adverse events compared with the HIV uninfected patients (HR = 4.0, 95% CI 1.5 – 10.5, *p* = 0.006), Figure 3.

In terms of medicines exposure, the DR-TB patients in our cohort were treated with individualized regimens, based on the susceptibility of the *M. tuberculosis* to specific second-line anti-TB medicines. In total, the patients were treated with 15 different second-line anti-TB medicines, while the HIV infected patients were additionally treated with HAART regimens, which consisted of lamivudine in combination with either zidovudine (AZT) or stavudine (d4T) and efavirenz (EFV) or nevirapine (NVP). None of the second-line anti-TB or antiretroviral medicines was statistically significantly associated with the occurrence of moderate-to-severe adverse events (Table 3). However, amikacin, ciprofloxacin and ethambutol tended to have a much higher risk when compared with the other second-line anti-TB medicines.

**Table 3:** **Use of specific second-line anti-TB or antiretroviral medicines and the risk of moderate-to-severe adverse events**

Anti-TB medicine	Number (%) treated with the medicine, N = 57	Univariate risk ratios	95% confidence interval	***p***-value
Amikacin	21 (36.8%)	1.5	0.8 – 2.6	0.18
Amoxicillin/Clavulanate	1 (1.8%)	0.0	-	0.36
Capreomycin	4 (7%)	1.1	0.4 – 3.1	0.86
Ciprofloxacin	19 (33.3%)	1.5	0.8 – 2.5	0.19
Clofazimine	1 (1.8%)	0	-	0.36
Cycloserine	27 (47.4%)	0.8	0.5 – 1.5	0.49
Ethambutol	35 (61.4%)	1.7	0.9 – 3.4	0.10
Ethionamide	52 (91.2%)	0.7	0.3 – 1.6	0.50
Isoniazid	4 (7%)	1.1	0.4 – 3.1	0.86
Kanamycin	30 (52.6%)	0.8	0.4 – 1.4	0.37
Levofloxacin	37 (64.9%)	0.6	0.4 – 1.1	0.11
Para aminosalicylic acid (PAS)	5 (8.8%)	1.4	0.6 – 2.9	0.50
Pyrazinamide	53 (93%)	0.9	0.3 – 2.5	0.86
Rifampicin	13 (22.8%)	0.8	0.4 – 1.7	0.56
Streptomycin	3 (5.3%)	0.7	0.1 – 3.6	0.66
Any HAART regimen	13 (22.8%)	0.8	0.4 – 1.7	0.56
Zidovudine (AZT)	5 (8.8%)	0.4	0.1 – 2.5	0.23
Stavudine (d4T)	6 (10.5%)	1.1	0.5 – 2.6	0.82
Efavirenz (EFV)	10 (17.5%)	0.9	0.4 – 1.9	0.70
Nevirapine (NVP)	3 (5.3%)	0.7	0.1 – 3.7	0.66

We further explored the association between the occurrence of moderate-to-severe adverse events, the specific medical interventions made to manage them and their specific outcomes. From a univariate analysis on the entire cohort, we found that moderate-to-severe adverse events determined whether the clinician chose to reduce the dose or to stop a specific DR-TB medicine, the risk ratio (RR) for the association being 3.8 (95% CI 1.2-11.8, *p* = 0.01). Upon stratification to assess for confounding or effect modification by HIV infection status, the association remained similar between HIV infected and HIV uninfected patients (RR = 4.2 and RR = 4.1 respectively) (Table 4).

**Table 4:** **Relationship between occurrence of moderate-to-severe AEs, medical actions to manage the AEs and their outcome**

		Stratified analysis	
Medical actions taken to manage AEs and outcome of AEs	Entire cohort analysis	HIV positive stratum	HIV negative stratum
Dose reduced or medicine stopped	*3.8 (1.2-11.8)*	4.2 (0.6-28.8)	*4.1 (1.02-16.2)*
	*p* = 0.007	*p* = 0.09	*p* = 0.04
Adjunctive therapy to treat AE symptoms	1.2 (0.5-2.7)	1.1 (0.4-2.8)	0.8 (0.1-6.0)
	*p* = 0.74	*p* = 0.61	*p* = 0.67
Regimen changed	0.5 (0.2-1.8)	0.5 (0.1-2.6)	0.6 (0.1-4.3)
	*p* = 0.31	*p* = 0.37	*p* = 0.50
Adverse events lasting ≥ 3 months	*3.1 (1.4-7.2)*	*3.6 (1.03-12.5)*	1.9 (0.5-7.2)
	*p* = 0.002	*p* = 0.009	*p* = 0.33

Numbers represent risk ratio (RR) point estimates, their corresponding 95% confidence intervals in brackets, and *p*-values; HIV = human immunodeficiency virus; AEs = adverse events.

There were HIV stratum-specific differences in the connection between occurrence of moderate-to-severe adverse events and those that lasted for three or more months. The risk ratios were RR = 3.6 (95% CI 1.03-12.5, *p* = 0.009) for the HIV infected sub-group versus RR = 1.9 (95% CI 0.5-7.2, *p* = 0.33) for the HIV uninfected one, demonstrating effect modification by HIV infection status (Table 4).

On the contrary, the occurrence of moderate-to-severe adverse events was not a determinant of the clinician’s decision to prescribe adjunctive medicines for certain adverse events (RR = 1.2, 95% CI 0.5-2.7, *p* = 0.74) or to change the entire DR-TB treatment regimen (RR = 0.5, 95% CI 0.2-1.8, *p* = 0.31) as shown in Table 4.

## Discussion

We found a high occurrence (89%) of any adverse event during DR-TB treatment. Similar findings have been reported elsewhere in the study by Koju *et al*. in Nepal who found that 80% of patients experienced at least one adverse event during treatment of tuberculosis [10]. Likewise, Leimane and co-researchers reported that 86% of patients in their study in Latvia experienced an adverse event, [11] while Bloss *et al*. reported an adverse event frequency of 79% in the same country [12]. Also, Shin *et al.*, have reported that 73% of MDR-TB patients in their Russian cohort experienced at least one adverse event [13]. This clearly shows that second-line anti-TB medicines are associated with a high frequency of adverse events.

The cumulative incidence of moderate-to-severe adverse events in our cohort was 46 events in 100 DR-TB patients. This finding is similar to that of Lanternier *et al.*, who reported an incidence of severe adverse events of 45.2 ± 11.3 per 100 person-years [14]. Such a high incidence of moderate-to-severe adverse events is a cause for concern for DR-TB programme managers, patients and clinicians.

In the present study, HIV co-infected DR-TB patients experienced more moderate-to-severe adverse events compared with the HIV uninfected patients (58 versus 20 events, *p* = 0.02). The HIV co-infected patients had a four-fold risk of experiencing moderate-to-severe adverse events compared with the HIV uninfected patients (HR = 4.0; 95% CI 1.5 – 10.5, *p =* 0.006). Similar findings have been reported by other researchers. For example, in Lima, Peru, Chung-Delgado *et al*. found that HIV infection increased the risk of adverse events during TB therapy by 3.45 (95% CI 1.61-7.45) [5]. Similarly, Lanternier *et al.* found that HIV infection increased the risk of TB treatment-associated adverse events by 3.9 (95% CI 2.1-7.5) [14]. Therefore, we urge clinicians to be more vigilant and to look out for potential moderate-to-severe adverse events when treating HIV co-infected DR-TB patients. This will help clinicians to identify adverse events early enough so that appropriate measures could be taken to mitigate them.

None of the second-line anti-TB or antiretroviral medicines was statistically significantly associated with the occurrence of moderate-to-severe adverse events. This was rather surprising as second-line anti-TB medicines are known to elicit moderate-to-severe adverse events [13, 15]. We argue that the failure to detect any statistically significant associations may have arisen from the low power of the study, rather than from a real biological difference in the way the medicines acted in the patients included in our study. However, amikacin, ciprofloxacin and ethambutol seemed to have a much higher risk than the other second-line anti-TB medicines. These three medicines tended to be prescribed together as part of a DR-TB regimen. This observation needs to be further investigated in a more powered and appropriately designed study that is capable of ruling out bias and confounding, for example, confounding by co-medication and confounding by indication of the medicines used for the treatment of DR-TB infection and concomitant HIV infection.

The frequency of a clinician reducing the dose or stopping the suspected offending medicine was 29%, while that of completely changing the treatment regimen was 18%. These findings are comparable with those of Prasad *et al.* where 21% of the patients developed major adverse events that required stoppage or change of the offending medicines [16]. Similarly, Bloss *et al.* have reported dosage reduction in 20% of the patients treated for MDR-TB [12]. On the other hand, Torun *et al.* reported a higher rate (55%) of withdrawal or discontinuation of second-line medicines during MDR-TB treatment [15]. As such, we advocate for clinicians to always consider reducing the dose, discontinuing or substituting the suspected offending medicine when managing moderate-to-severe adverse events during the treatment of DR-TB.

Moderate-to-severe adverse events were the main reason for clinicians’ decision to either reduce the dose or to stop a specific DR-TB medicine (RR = 3.8, 95% CI 1.2-11.8, *p* = 0.01). This remained true, irrespective of the patients’ HIV infection status. However, patients co-infected with HIV tended to suffer more from adverse events that lasted for three or more months (RR = 3.6, *p* = 0.009) compared with the HIV uninfected patients (RR = 1.9, *p* = 0.33). The reason for the longer duration of some adverse events in HIV co-infected patients is unclear, but we think that it could be related to the patients’ weakened immune status and to the pharmacological interactions between some of the anti-TB and antiretroviral medicines [2, 3]. Clinicians need to be alert that moderate-to-severe adverse events in patients on concomitant DR-TB and HIV treatment may potentially last for at least three months. Such persistence of moderate-to-severe adverse events could negatively impact the patients’ ability to adhere to both treatments, possibly compromising patients’ DR-TB and HIV treatment outcomes.

### Study limitations and strengths

The adverse events described in our study were symptomatic and were clinician or patient-reported. The over- or under-reporting of some of the adverse events, especially those that require objective confirmatory tests, may have biased the data. Furthermore, since no causality assessments were done, it was not always possible to attribute particular adverse events to a specific medicine at the individual patient level. Despite this limitation, we were able to reveal the magnitude and nature of the association between moderate-to-severe adverse events and HIV co-infection. We were also able to elucidate on the relationship between the occurrence of moderate-to-severe adverse events and the various medical interventions made to manage the adverse events as well as the outcomes of these adverse events. By unravelling some of the complexities of DR-TB treatment, this study contributes to the epidemiology of adverse events in DR-TB treatment, hence enriching the evidence base upon which clinicians and TB programme managers may use to make decisions on improving treatment of DR-TB infection.

## Conclusions

Moderate-to-severe adverse events are common during DR-TB treatment. They are more likely to occur and to persist in HIV co-infected patients than in HIV uninfected ones. Clinicians may alleviate the discomfort and reduce the harm of such adverse events by reducing the dose, stopping or by changing the suspected offending medicine. Managers of TB control programs should strengthen pharmacovigilance systems so that clinically important adverse events could be detected early and control or mitigation measures instituted in time, for example, through revision of treatment guidelines. We recommend a larger study to generate more precise and conclusive findings on the determinants of the moderate-to-severe adverse events and the effect of the events on DR-TB treatment outcomes and patients’ health-related quality of life.

## Electronic supplementary material

Additional file 1: **Annexure 17.** MDR-TB patient treatment card, MoHSS. (PDF 692 KB)
