# Supplementary material for: Occurrence and clinical management of moderate-to-severe adverse events during drug-resistant tuberculosis treatment: a retrospective cohort study
Source: J Pharm Policy Pract. 2014 Oct 21;7(1):14. doi: 10.1186/2052-3211-7-14 (PMC4219090; doi:10.1186/2052-3211-7-14)
Supplement: Supplementary file 1 — Additional file 1: Annexure 17. MDR-TB patient treatment card, MoHSS. (PDF 692 KB) [file 40545_2014_22_MOESM1_ESM.pdf]

Annexure 17

MDR-TB patient treatment card

Ministry of Health and Social Services — Republic of Namibia — Code XXXX

National Tuberculosis Control Programme

MDR-TB PATIENT TREATMENT CARD (front side)

**MDR-TB Treatment Unit:**	**Registration number:**	**Date of registration:**		
**Patient name:**		**Age**	M	F
**Address:**		**Telephone number:**		
**Employment/profession**	**Mother tongue:**			

**Previous TB treatment history:**	Number of previous treatments with first-line TB drugs (≥ 4 weeks):	
	Number of previous treatments with second-line TB drugs (≥ 4 weeks):	

**Period TB drugs were taken** (month/years)			**Diagnostic category (tick)**			
Rifampicin (R)			New patient, never treated for TB, or treated for less than 4 weeks			
Isoniazid (H)						
Pyrazinamide (Z)			Previously treated with first-line drugs more than 4 weeks			
Ethambutol (E)						
Streptomycin (S)			Previously treated with second-line drugs more than 4 weeks			
Amikacin (Am)						
Ciprofloxacin (Ci)			**HIV test result** (tick)	Positive	Negative	Not done
Ethionamide (Et)						

**Pulmonary TB**	**Extra-Pulmonary TB**		**Body weight** (kgs)
**Initial sputum-smear results** (date, lab. Number) (neg, positive and grading, not done, no data)	1	2	3
**Initial Culture results** (date, lab number) Negative/positive *M.tb*/contaminated/not done/pending	1	2	
**Initial Drug Sensitivity Test:** (Date collected, laboratory number, date result) (Res, Sens, not done, pending)	1	2	

Close contacts:

(Left inner side)

Medical history:
(Adverse reactions and allergies to non-TB medications; last menstrual period; method of contraception; pregnancy history)

Other complicating conditions:
(Diabetes, renal insufficiency, hepatitis, drug or alcohol abuse, psychiatric disorders, depression etc.)

Other drugs that the patient is currently taking:

Physical examination:
(General physical condition, blood pressure, length, BMI, full physical examination, urine analysis, liver/kidney function)

X-ray findings:

TREATMENT (Right inner side)

**Date treatment started**				
	**Initial phase**	**Dose**	**Continuation phase**	**Dose**
Standard second-line	Amikacin		Ethionamide	
	Ethionamide		Ciprofloxacin	
	Ciprofloxacin		Ethambutol	
	Pyrazinamide			
	Ethambutol			
Specific regimen (DST result)	Amikacin		-	
	Ethionamide		Ethionamide	
	Ciprofloxacin		Ciprofloxacin	
	Cycloserin		Cycloserin	
	Pyrazinamide		Pyrazinamide	
	Ethambutol		Ethambutol	
	Rifampicin		Rifampicin	
	Streptomycin		-	
	Isoniazid		Isoniazid	
Additional treatment	Cotrimoxazole		Cotrimoxazole	
	Pyridoxine		Pyridoxine	
Anti-Retroviral Treatment				
Other medicines				

**MONITORING (Treatment duration in months)**	**Sputum-smear**			**Culture (always 2 specimens)**	**DST (See Guidelines)**	**Body weight Kg**
	1	2	3			
Pre-treatment						
2						
3						
4						
5						
6						
9						
12						
15						
18						
21						
24						

INITIAL PHASE (Write date after all drugs were swallowed) (Back)

Week	Mon	Tues	Wed	Thurs	Frid	Sat	Sun

CONTINUATION PHASE (loose in lay form) Page 1

Week	Mon	Tues	Wed	Thurs	Frid	Sat	Sun

Annexure 18

MDR-TB drug side effect monitoring form

Ministry of Health and Social Services — Republic of Namibia — National Tuberculosis Control Programme

MDR-TB DRUG SIDE EFFECT MONITORING FORM

INTENSIVE PHASE

Adverse effect (indicate grading*)	Week																								Management	Outcome
	1	2	3	4	5	6	7	8	9	10	11	12	13	14	15	16	17	18	19	20	21	22	23	24		
Abdominal pain																										
Constipation																										
Decreased hearing																										
Depression																										
Diarrhea																										
Dizziness																										
Fatigue																										
Fever																										
Headache																										
Joint pain																										
Nausea																										
Psychosis																										
Rash																										
Skin colourisation																										
Tinnitus																										
Tremors																										
Vision changes																										
Vomiting																										
Other (list)																										

* Grading: 1 = mild; requiring no intervention   2 = moderate; requiring palliative intervention   3 = severe; requiring change in treatment

Ministry of Health and Social Services | Republic of Namibia | National Tuberculosis Control Programme

MDR-TB DRUG SIDE EFFECT MONITORING FORM

CONTINUATION PHASE

Adverse effect (indicate grading*)	Month **																				Management	Outcome
Abdominal pain																						
Constipation																						
Decreased hearing																						
Depression																						
Diarrhea																						
Dizziness																						
Fatigue																						
Fever																						
Headache																						
Joint pain																						
Nausea																						
Psychosis																						
Rash																						
Sink colourisation																						
Tinnitus																						
Tremors																						
Vision changes																						
Vomiting																						
Other (list)																						

* Grading: 1 = mild; requiring no intervention 2 = moderate; requiring palliative intervention 3 = severe; requiring change in treatment
** Indicate in the first column the month of treatment that continuation phase started
